# Application of whole exome sequencing in the diagnosis of muscular disorders: a study of Taiwanese pediatric patients

**DOI:** 10.3389/fgene.2024.1365729

**Published:** 2024-05-15

**Authors:** Chung-Lin Lee, Chih-Kuang Chuang, Huei-Ching Chiu, Ya-Hui Chang, Yuan-Rong Tu, Yun-Ting Lo, Hsiang-Yu Lin, Shuan-Pei Lin

**Affiliations:** ^1^ Department of Pediatrics, MacKay Memorial Hospital, Taipei, Taiwan; ^2^ Institute of Clinical Medicine, National Yang-Ming Chiao-Tung University, Taipei, Taiwan; ^3^ Department of Rare Disease Center, MacKay Memorial Hospital, Taipei, Taiwan; ^4^ Department of Medicine, Mackay Medical College, Taipei, Taiwan; ^5^ Mackay Junior College of Medicine, Nursing and Management, Taipei, Taiwan; ^6^ Division of Genetics and Metabolism, Department of Medical Research, MacKay Memorial Hospital, Taipei, Taiwan; ^7^ College of Medicine, Fu-Jen Catholic University, Taipei, Taiwan; ^8^ Department of Medical Research, China Medical University Hospital, China Medical University, Taichung, Taiwan; ^9^ Department of Infant and Child Care, National Taipei University of Nursing and Health Sciences, Taipei, Taiwan

**Keywords:** muscular disorders, muscular dystrophies, congenital myopathies, whole exome sequencing, mutational diversity

## Abstract

**Background:**

Muscular dystrophies and congenital myopathies encompass various inherited muscular disorders that present diagnostic challenges due to clinical complexity and genetic heterogeneity.

**Methods:**

This study aimed to investigate the use of whole exome sequencing (WES) in diagnosing muscular disorders in pediatric patients in Taiwan. Out of 161 pediatric patients suspected to have genetic/inherited myopathies, 115 received a molecular diagnosis through conventional tests, single gene testing, and gene panels. The remaining 46 patients were divided into three groups: Group 1 (multiplex ligation-dependent probe amplification–negative Duchenne muscular dystrophy) with three patients (6.5%), Group 2 (various forms of muscular dystrophies) with 21 patients (45.7%), and Group 3 (congenital myopathies) with 22 patients (47.8%).

**Results:**

WES analysis of these groups found pathogenic variants in 100.0% (3/3), 57.1% (12/21), and 68.2% (15/22) of patients in Groups 1 to 3, respectively. WES had a diagnostic yield of 65.2% (30 patients out of 46), detecting 30 pathogenic or potentially pathogenic variants across 28 genes.

**Conclusion:**

WES enables the diagnosis of rare diseases with symptoms and characteristics similar to congenital myopathies and muscular dystrophies, such as muscle weakness. Consequently, this approach facilitates targeted therapy implementation and appropriate genetic counseling.

## Introduction

### Background

Muscular dystrophies (MD) and congenital myopathies (CM) consist of a wide variety of inherited muscle disorders that lead to significant disability in patients of different age groups (North et al., 2014; Mercuri et al., 2019). MD feature progressive loss of muscle strength along with abnormal pathological changes seen on muscle biopsy (Mercuri et al., 2019). They have traditionally been categorized based on age at onset, main clinical and biopsy characteristics, and results of immunostaining (Sewry et al., 2019). MD include conditions affecting proteins like dystrophin, sarcoglycans and dysferlin. Identifying the defective proteins can be challenging as multiple proteins may be affected. CM are a group of muscle diseases present from birth, usually causing hypotonia and weakness (North et al., 2014). They tend to be static or slowly progressive. CM have been classified according to the predominant morphological features seen on muscle biopsy (Witting et al., 2017).

MD are a heterogeneous group of inherited disorders characterized by progressive muscle weakness and degeneration. The most common types of MD include Duchenne muscular dystrophy (DMD), Becker muscular dystrophy (BMD), limb-girdle muscular dystrophies (LGMD), facioscapulohumeral muscular dystrophy (FSHD), and congenital muscular dystrophies (CMD) (Mercuri et al., 2019). DMD and BMD are caused by mutations in the dystrophin gene (DMD) and are characterized by progressive proximal muscle weakness, with DMD being more severe than BMD (Birnkrant et al., 2018). LGMD represents a group of disorders with predominant weakness in the shoulder and pelvic girdle muscles, caused by mutations in various genes encoding proteins involved in muscle function and structure (Straub et al., 2018). FSHD is an autosomal dominant disorder caused by contractions in the D4Z4 repeat region on chromosome 4q35, leading to characteristic facial and shoulder girdle muscle weakness (Tawil et al., 2015). CMDs are a group of early-onset muscular dystrophies with variable severity and genetic heterogeneity (Kang et al., 2015).

Thanks to significant advances in molecular genetics during the last 2 decades, molecular genetic testing has dramatically improved the diagnosis of neuromuscular disorders (North et al., 2014; Mercuri et al., 2019). Over 100 genes associated with neuromuscular disorders have been identified (Benarroch et al., 2020). The ability to correlate specific gene defects to clinical phenotypes has transformed our understanding of these conditions. Clinical manifestations of neuromuscular disorders in pediatric patients often lack specificity, presenting nonspecific symptoms, such as motor developmental delay, hypotonia, and weakness, which are inadequate for a definitive diagnosis. Many neuromuscular diseases have phenotypic heterogeneity, which makes diagnosis challenging. Next-generation sequencing (NGS) in the molecular diagnosis of neuromuscular diseases is becoming more common. The identification of causative genes may be achieved with enhanced efficiency by integrating the patient’s phenotype, pathological findings from muscle biopsy, and sequencing data (Böhm et al., 2013; Valencia et al., 2013). However, costs remain a barrier to implementing NGS technology in many settings.

Whole exome sequencing (WES) has emerged as a powerful diagnostic tool for patients with suspected genetic disorders during the last decade. WES focuses on gene protein-coding regions, allowing for comprehensive analysis of variants across many genes simultaneously. WES has been found to have a diagnostic yield of 25%–73% in pediatric patients with neuromuscular disorders (Richards et al., 2015). However, some variation is based on the patient cohort and the filtering strategies. WES enables genetic diagnosis even in patients with atypical symptoms or ultra-rare conditions.

The primary aim of this study was to investigate the diagnostic yield and clinical utility of WES in a cohort of Taiwanese pediatric patients with suspected CM or MD. We sought to determine the proportion of patients who received a genetic diagnosis through WES and to characterize the spectrum of genetic variants identified in this population. Furthermore, we aimed to explore the potential of WES to refine clinical diagnoses, guide management decisions, and inform genetic counseling for affected families.

## Research design

### Study design and patient population

**FIGURE 1:**
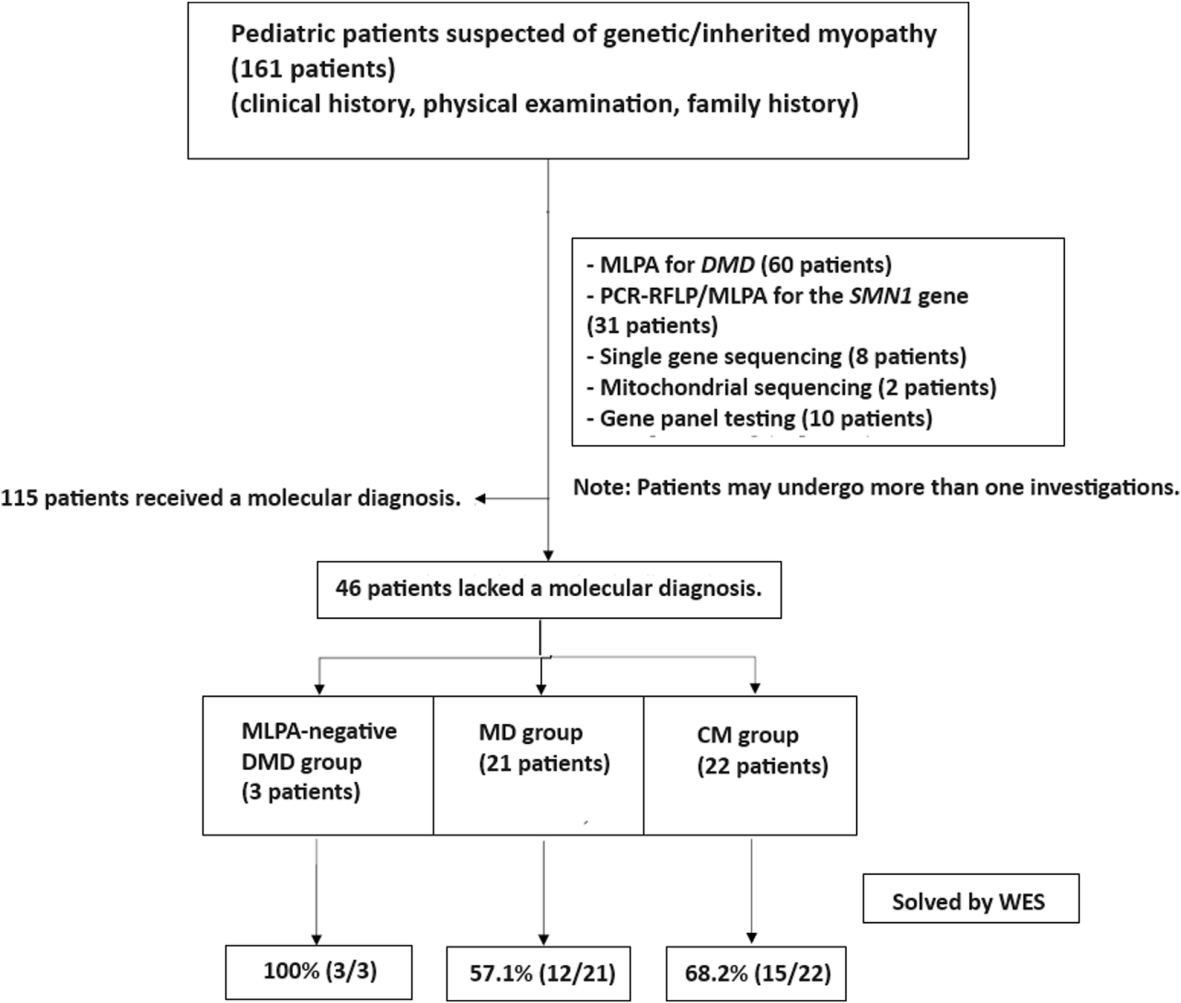
Patient enrollment workflow. Out of 161 patients with suspected genetic myopathies, 115 received a molecular diagnosis through conventional tests, single gene testing, and gene panels. MLPA for DMD and SMA identified causative variants in 60 and 31 patients, respectively. Other single gene tests and gene panels established a diagnosis in 18 patients. The remaining 46 patients underwent whole exome sequencing (WES) and were categorized into three groups: MLPA-negative DMD (*n* = 3), muscular dystrophies (MD; *n* = 21), and congenital myopathies (CM; *n* = 22).

This study adhered to the principles of the Helsinki Declaration. From January 2018 to August 2023, a descriptive, cross-sectional study was conducted on pediatric patients clinically diagnosed with MD and CM at MacKay Memorial Hospital in Taipei, Taiwan. MD and CM were clinically diagnosed by experienced pediatric neurologists based on medical histories, age of symptom onset, disease progression, physical examinations, and standard diagnostic tests. Patients with suggestive clinical phenotypes underwent single gene testing, including multiplex ligation-dependent probe amplification (MLPA) for spinal muscular atrophy (*SMN1*) and Duchenne muscular dystrophy (*DMD*). After preliminary studies, patients with no known molecular etiologies were recruited for WES analysis (Figure 1).

In our study, none of the 46 patients who underwent WES had a prior muscle biopsy. The decision to proceed directly to WES was made by the treating physicians based on a thorough clinical evaluation and the suspicion of a genetic etiology for the patients’ muscular disorders. This approach was aimed at streamlining the diagnostic process and minimizing invasive procedures for the patients.

The decision to segregate the MD and DMD groups was based on the distinct clinical and molecular characteristics of these conditions. DMD is a well-characterized X-linked disorder caused by mutations in the *DMD* gene, whereas the MD group encompasses a heterogeneous set of disorders with varying genetic etiologies and clinical presentations.

As for the MLPA-negative DMD group, these patients had initially undergone MLPA testing for the *DMD* gene, which did not identify any causative variants. However, given the strong clinical suspicion of DMD, they were included in our cohort to explore the potential of WES in detecting single nucleotide variants or small indels that may have been missed by MLPA.

Regarding the MD group, these patients did not receive MLPA, single-gene sequencing, or gene panel analysis prior to WES. They were included in our cohort based on the clinical diagnosis of muscular dystrophies but without a confirmed molecular etiology. The decision to proceed directly to WES for these patients was made to streamline the diagnostic process and avoid sequential testing, which can be time-consuming and costly.

### DNA extraction, exome sequencing and bioinformatic analysis

Genomic DNA was extracted from peripheral blood samples using the QIAamp DNA Blood Mini Kit (Qiagen, Hilden, Germany) according to the manufacturer’s instructions. Exome capture was performed using the SureSelect Human All Exon V5 Kit (Agilent Technologies, Santa Clara, CA, United States), which targets approximately 50 Mb of the human exonic regions. Sequencing libraries were prepared using the SureSelect XT Library Prep Kit (Agilent Technologies) and sequenced on the Illumina HiSeq 4000 platform (Illumina, San Diego, CA, United States) with 150-bp paired-end reads.

Variants were filtered based on the following criteria: (a) located in exons or flanking introns, (b) resulting in amino acid changes, (c) minor allele frequency less than 1% in 1000 Genomes database, (d) allele frequency less than 0.1% in Genome Aggregation Database (gnomAD), (e) missense variants predicted to be deleterious by SIFT and PolyPhen algorithms, and (f) variants associated with neuromuscular phenotypes.

Gene variants linked to neuromuscular diseases were first analyzed, followed by classification of potential variants by clinical geneticists and neurologists based on American College of Medical Genetics and Genomics guidelines (Straub et al., 2018). Variants were considered “novel” if previously unreported in PubMed database.

Cases were categorized as “solved” if pathogenic or likely pathogenic variants were identified in phenotype-associated genes with matching inheritance patterns. Cases with only one variant associated with recessive disease were designated as “partially solved”.

## Results

**FIGURE 2:**
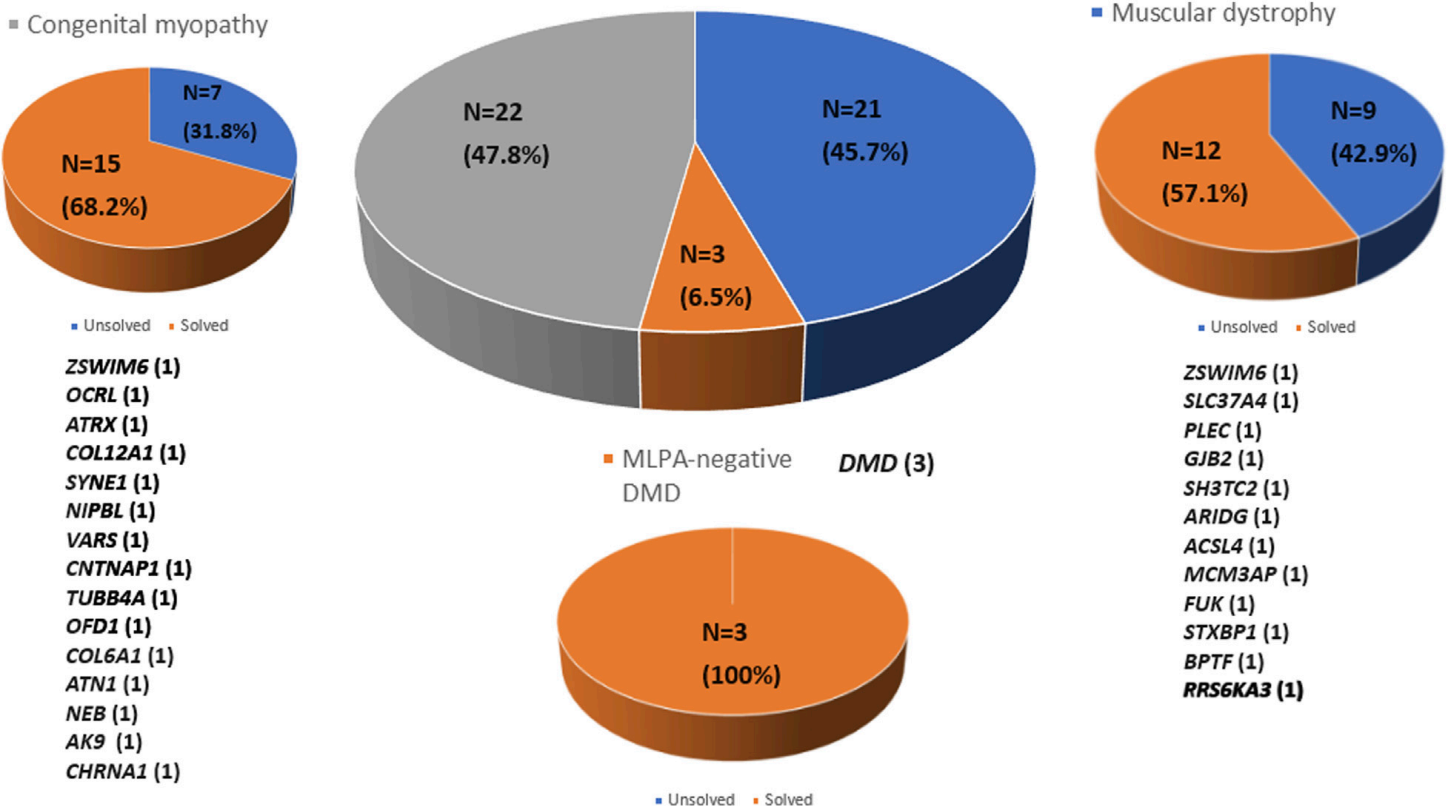
Molecular diagnosis rate by disease group using whole exome sequencing. Molecular diagnoses were achieved in 65.2% of all patients analyzed (*n* = 30). The number of patients diagnosed in each disease group is indicated in parentheses.

Whole exome sequencing (WES) was conducted on 46 patients from 46 unrelated families. Among these, 59% (27/46) were males. Onset of disease ranged from birth to 37 years, with a median of 5.9 years (Table 1). Three patients (6.5%) tested negative for Duchenne muscular dystrophy (DMD) on multiplex ligation-dependent probe amplification (MLPA). Twenty-one patients (45.7%) were diagnosed with other muscular dystrophies (MD), while 22 patients (47.8%) had congenital myopathies (CM) (Figure 1). Trio-based WES was undertaken for 14 patients (30.4%) from 14 families. Overall, disease-causing variants were uncovered in 65.2% (30/46) of patients (Figure 2).

**TABLE 1:** Demographic data.

	Frequency
Sex, N (%)	
Male	27 (58.7%)
Female	19 (41.3%)
Age, median (range)	
Current age	5Y9M (0–31Y)
Age of onset	20M (0–10Y)
Age at clinical diagnosis	7Y6M (0–31Y)
Clinical diagnosis, N (%)	
Congenital myopathies	22 (47.8%)
muscular dystrophies	24 (52.2%)

### The CM group

In a cohort of 22 patients with CM, WES successfully provided a genetic diagnosis in 15 patients (68.2%). Among these 15 solved cases, six patients (40.0%) had autosomal recessive variants, another six patients (40.0%) had *de novo* variants, and the remaining three patients (20.0%) had X-linked variants. In total, 15 distinct pathogenic variants were identified, each located in a different gene as follows: *ZSWIM6, OCRL, ATRX, COL12A1, SYNE1, NIPBL, VARS, CNTNAP1, TUBB4A, OFD1, COL6A1, ATN1, NEB, AK9*, and *CHRNA1* (n = 1 variant per gene). This study demonstrates the use of WES in diagnosing genetically heterogeneous conditions such as CM by allowing the discovery of causal variants across many genes (Table 2).

**TABLE 2:** Gene analysis of congenital myopathies (CM) group.

Gene	Number of patients	Variants	ACMG classification	OMIM phenotype	Consistent with gene
ZSWIM6	1	c.532_533insT, p.Ala178Valfs*76	Likely pathogenic	Neurodevelopmental disorder with movement abnormalities and/or seizures	No
OCRL	1	c.1174 + 1G>T	Likely pathogenic	Lowe syndrome/Dent disease 2	No
ATRX	1	c.736C>T, p.Arg246Cys	Likely pathogenic	Alpha-thalassemia/mental retardation	No
COL12A1	1	c.5894G>A, p.Gly1965Glu	Likely pathogenic	Bethlem myopathy 2/Ullrich congenital muscular dystrophy	Yes
SYNE1	1	c.5627G>A, p.Ser1876Asn	Likely pathogenic	SYNE1-related autosomal recessive cerebellar ataxia/Emery-Dreifuss muscular dystrophy 4	Yes
NIPBL	1	c.53G>C, p.Ser18Thr	Likely pathogenic	Cornelia de Lange syndrome 1	No
VARS	1	c.3596G>A, p.Arg1199Gin	Likely pathogenic	Neurodevelopmental disorder with microcephaly and seizures	No
CNTNAP1	1	c.3361C>T, p.Arg1121*	Pathogenic	Congenital hypomyelinating neuropathy	Yes
TUBB4A	1	c.1172G>A, p.Arg391His	Likely pathogenic	Hypomyelinating leukodystrophy	No
OFD1	1	c.2del, p.Met1	Likely pathogenic	Joubert syndrome 10/Orofaciodigital syndrome I	No
COL6A1	1	c.850G>A, p.Gly284Arg	Likely pathogenic	Bethlem myopathy 1/Ullrich congenital muscular dystrophy	Yes
ATN1	1	c.1449C>A, p.His483Gln	Likely pathogenic	Dentatorubral-pallidoluysian atrophy	No
NEB	1	c.11606T>C, p.Tyr3869Tyr	Likely pathogenic	Nemaline myopathy 2	Yes
		c.18800T>C, p.Ile6267Thr			
AK9	1	c.3614G>T, p.Arg1205Ile	Likely pathogenic	Arthrogryposis multiplex congenita	No
		c.529G>T, p.Asp177Tyr			
CHRNA1	1	c.257G>A, p.Arg86His	Pathogenic	Myasthenic syndrome, congenital, 1B	Yes

### The MD group

WES enabled genetic diagnosis in 12 (57.1%) of 21 patients with MD. Among the 12 solved cases, autosomal recessive inheritance was identified in seven patients (58.3%), *de novo* variants in four patients (33.3%), and X-linked inheritance in one patient (8.4%). A total of 12 pathogenic variants were detected, each in a different gene as follows: *ZSWIM6, SLC37A4, PLEC, GJB2, SH3TC2, ARIDG, ACSL4, MCM3AP, FUK, STXBP1, BPTF,* and *PRS6KA3* (*n* = 1 for each) (Table 3).

**TABLE 3:** Gene analysis of muscular dystrophies (MD) group.

Gene	Number of patients	Variants	ACMG classification	OMIM phenotype	Consistent with gene
ZSWIM6	1	c.481delC, p.Ala161fs	VUS	Neurodevelopmental disorder with movement abnormalities and/or seizures	No
SLC37A4	1	c.1042_1043delCT, p.Leu348fs*53	VUS	Glycogen storage disease Ib	No
		c.898C>T, p.Arg300Cys			
PLEC	1	c.9343C>T, p.Arg3115Cys	VUS	Epidermolysis bullosa simplex with muscular dystrophy	Yes
		c.13192G>A, p.Ala4398Thr			
GJB2	1	c.109G>A, p.Val37Ile	Pathogenic	Bart-Pumphrey syndrome	No
SH3TC2	1	c.1817_1818del, p.Glu606ValfsTer2	Likely pathogenic	Charcot-Marie-Tooth disease, type 4C	No
ARIDG	1	c.1717dup, p.Trp573LeufsTer45	Pathogenic	Coffin-Siris syndrome 1	No
ACSL4	1	c.1126–4T>C	VUS	Intellectual developmental disorder	No
MCM3AP	1	c.5383G>A, p.Glu1795Lys	Likely pathogenic	Leukodystrophy, hypomyelinating, 5	No
		c.998G>T, p.Cys333Phe			
FUK	1	c.428C>T, p.Pro143Leu	Likely pathogenic	Congenital disorder of glycosylation with defective fucosylation	No
		c.1341 + 1G>T			
STXBP1	1	c.1706C>G, p.Ser569Cys	Likely pathogenic	Developmental and epileptic encephalopathy 4	No
BPTF	1	c.205G>C, p.Gly69Arg	Likely pathogenic	Neurodevelopmental disorder with dysmorphic facies and distal limb anomalies	No
RPS6KA3	1	c.2182C>T, p.Gln728*	Pathogenic	Coffin-Lowry syndrome	No

### The MLPA-negative DMD group

Whole exome sequencing revealed three pathogenic variants in the DMD gene in the MLPA-negative DMD group, confirming the diagnosis of DMD. These variants included two missense mutations and one nonsense mutation (Table 4).

**TABLE 4:** Gene analysis of MLPA negative DMD group.

Gene	Number of patients	Variants	ACMG classification	OMIM phenotype	Consistent with gene
DMD	3	c.7354G>T, p.Glu2452*(E2452*)	Pathogenic	Duchenne muscular dystrophy	Yes
		c.7993A>G, p.Asn2665Asp			
		c.5190G>C, p.Lys1730Asn			

In this study, WES revealed causative variants in 65.2% (30/46) of our cohort. The diagnostic yield was 100% (3/3) in the MLPA-negative DMD group, 57.1% (12/21) in the MD group, and 68.2% (15/22) in the CM group (Figure 2). The most commonly mutated gene was *ZSWIM6*, implicated in neurodevelopmental disorder with movement abnormalities, abnormal gait, and autistic features. Other recurrently affected genes included DMD in the MD group and *SYNE1, ATRX*, and *COL6A1* in the CM group. The key clinical and molecular findings of the genetically diagnosed patients are summarized in Table 2, Table 3 and Table 4.

In our cohort, we identified several *de novo* mutations in patients whose genetic findings revealed a diagnosis different from the initial clinical suspicion of a muscular disorder. Specifically, in the congenital myopathies group, 6 patients (27.3%) harbored *de novo* variants in the following genes: *ZSWIM6, OCRL, ATRX, SYNE1, NIPBL,* and *TUBB4A.* In the muscular dystrophies group, 4 patients (19.0%) had *de novo* variants in the genes *ARIDG, MCM3AP, STXBP1*, and *BPTF*.

The modes of inheritance observed for the identified mutations included autosomal dominant, autosomal recessive, and X-linked inheritance patterns. We found variants in 9 genes associated with autosomal dominant conditions (*COL12A1, NIPBL, TUBB4A, SYNE1, ARIDG, BPTF, STXBP1, ZSWIM6, GJB2*), 13 genes linked to autosomal recessive disorders (*VARS, CNTNAP1, COL6A1, NEB, AK9, CHRNA1, ATN1, SLC37A4, PLEC, SH3TC2, MCM3AP, FUK, COL12A1*), and 6 genes related to X-linked diseases (*OCRL, ATRX, OFD1, ACSL4, PRS6KA3, DMD*).

Out of the 30 patients with a genetic diagnosis, 10 patients (33.3%) had findings consistent with their initial clinical diagnosis of a neuromuscular disorder. However, in 20 patients (66.6%), the genetic testing revealed a different diagnosis, such as neurodevelopmental disorders (e.g., ZSWIM6-related disorder), deafness (GJB2-related), or Lowe syndrome (OCRL-related). These findings underscore the importance of genetic testing in refining clinical diagnoses and highlight the potential for phenotypic overlap between neuromuscular disorders and other neurological conditions.

To assess the potential functional impact of the identified variants, we performed a conservation analysis using the University of California Santa Cruz (UCSC) Genome Browser and the Conserved Domains Database (CDD) from the National Center for Biotechnology Information (NCBI). The conservation status of each variant is summarized in Supplementary Tables S1–S3. In the CM group, 60% (9/15) of the variants were located in highly conserved regions, while 20% (3/15) were in moderately conserved regions, and 13.3% (2/15) were in poorly conserved regions. One variant (OFD1 c.2del) could not be assessed for conservation as it affects the start codon. In the MD group, 50% (6/12) of the variants were in highly conserved regions, 41.7% (5/12) were in moderately conserved regions, and 8.3% (1/12) were in poorly conserved regions. All three variants identified in the MLPA-negative DMD group were located in highly conserved regions of the *DMD* gene. Overall, 60% (18/30) of the identified variants were found in highly conserved regions, suggesting that they may have a significant impact on protein function and disease pathogenesis.

To provide context for our findings, we reviewed the literature for previously reported mutations in the genes identified in our study (Supplementary Table S4). We found that several of the variants we detected had been described in earlier studies, providing further evidence for their pathogenicity. For example, the *DMD* gene variants c.7354G>T, c.7993A>G, and c.5190G>C, which we found in the MLPA-negative DMD group, have been reported in patients with Duchenne muscular dystrophy (Flanigan et al., 2009; Tuffery-Giraud et al., 2009; Okubo et al., 2016). Similarly, the *PLEC* gene variants c.9343C>T and c.13192G>A, identified in the MD group, have been associated with epidermolysis bullosa simplex with muscular dystrophies (Natsuga et al., 2010; Winter and Wiche, 2013). In the CM group, we identified the *COL12A1* variant c.5894G>A and the *COL6A1* variant c.850G>A, which have been previously reported in patients with Bethlem myopathy or Ullrich congenital muscular dystrophy (Lampe and Bushby, 2005; Butterfield et al., 2013; Naghipoor et al., 2023).

## Discussion

In this study, we performed WES on 46 patients from 46 unrelated Taiwanese families clinically diagnosed with MD or CM. Simultaneous analysis of their clinical phenotypes and WES data enabled a correlation between the sequencing results and other diagnostic modalities. WES had a diagnostic yield of 65.2% (30/46 patients). DMD was the most often implicated MD gene (10%; 3/30 diagnosed cases), whereas *ZSWIM6* was the most commonly identified disease-causing gene in MD and CM cases. The spectrum of implicated genes is consistent with previous pediatric studies on neuromuscular disorders using WES (Chae et al., 2015; Harris et al., 2017).

Our study demonstrates the feasibility and diagnostic yield of using WES as a first-tier test in a selected group of patients with suspected genetic muscular disorders. By proceeding directly to WES, we were able to provide a genetic diagnosis in 65.2% of patients without subjecting them to invasive procedures like muscle biopsy. This approach has the potential to reduce diagnostic delays and improve patient care in carefully selected cases. However, it is important to recognize that muscle biopsy remains a valuable diagnostic tool in cases with unclear genetic findings or when tissue-level information is needed to guide treatment decisions.

Our findings demonstrate the clinical utility of WES in the genetic diagnosis of pediatric neuromuscular disorders in a Taiwanese cohort. The overall diagnostic yield of 65.2% is consistent with previous studies reporting yields of 25%–73% (Chae et al., 2015; Richards et al., 2015; Todd et al., 2015; Harris et al., 2017; Waldrop et al., 2019; Babic-Bozovic et al., 2021). The high diagnostic rate in the MLPA-negative DMD group highlights the ability of WES to detect single nucleotide variants and small indels missed by MLPA or gene panels. In the MD and CM groups, WES enabled genetic diagnosis in over half of the patients, reflecting its effectiveness in resolving genetically heterogeneous disorders.

Our conservation analysis revealed that a significant proportion (60%) of the identified variants were located in highly conserved regions of their respective proteins. This finding underscores the potential functional importance of these residues and suggests that variants affecting these positions may have a greater impact on protein function and, consequently, disease pathogenesis. Highly conserved residues are more likely to be critical for maintaining protein structure, stability, and function, as they have been preserved throughout evolution due to their essential roles (Ng and Henikoff, 2003; Doniger et al., 2008). Variants in these regions may disrupt essential protein-protein interactions, catalytic sites, or other key functional domains, leading to deleterious effects on protein function (Miller and Kumar, 2001; Gao et al., 2018). In contrast, variants in poorly conserved regions may be better tolerated and have less severe consequences for protein function and disease manifestation (Ferrer-Costa et al., 2002; Bajaj et al., 2007). Our findings highlight the importance of considering evolutionary conservation when assessing the potential pathogenicity of genetic variants and prioritizing them for further functional studies. By integrating conservation data with other lines of evidence, such as clinical phenotypes and computational predictions, we can gain a more comprehensive understanding of the molecular basis of muscular disorders and improve our ability to interpret the clinical significance of genetic variants (Richards et al., 2015; Tavtigian et al., 2018).

Our literature review revealed that several of the variants identified in our study have been previously reported in patients with muscular disorders. In the MLPA-negative DMD group, we found three pathogenic variants in the *DMD* gene (c.7354G>T, c.7993A>G, and c.5190G>C) that have been described in patients with Duchenne muscular dystrophy (Flanigan et al., 2009; Tuffery-Giraud et al., 2009; Okubo et al., 2016). In the MD group, we identified two variants (c.9343C>T and c.13192G>A) in the *PLEC* gene, which has been implicated in epidermolysis bullosa simplex with muscular dystrophies (Natsuga et al., 2010; Winter and Wiche, 2013). Furthermore, in the CM group, we detected a likely pathogenic variant (c.5894G>A) in the *COL12A1* gene, known to be associated with Bethlem myopathy and Ullrich congenital muscular dystrophy (Naghipoor et al., 2023), as well as a likely pathogenic variant (c.850G>A) in the *COL6A1* gene, which causes Bethlem myopathy and Ullrich congenital muscular dystrophy (Lampe and Bushby, 2005; Butterfield et al., 2013). The consistency between our findings and previous reports strengthens the evidence for the pathogenicity of these variants and highlights the clinical relevance of the genes identified in our study. These results underscore the utility of WES in identifying clinically relevant mutations in patients with muscular disorders and contribute to our understanding of the genetic basis of these conditions.

Interestingly, the *ZSWIM6* gene was implicated in both the CM and MD groups in our study. The *ZSWIM6* gene has been associated with neurodevelopmental disorder with movement abnormalities and/or seizures, a condition characterized by a wide range of clinical manifestations, including intellectual disability, seizures, and abnormal movements (Twigg et al., 2016; Palmer et al., 2017). The two patients in our study with ZSWIM6 variants presented with overlapping features of muscle weakness and developmental delay, despite being classified into different disease categories (CM and MD) based on their initial clinical assessment. This finding highlights the limitations of relying solely on clinical symptoms to differentiate between CM and MD, as there can be significant phenotypic overlap between these conditions. Our results underscore the importance of genetic testing, particularly WES, in accurately diagnosing and categorizing patients with complex neuromuscular disorders.

Previous studies using WES for pediatric neuromuscular disorders found diagnostic yields ranging from 37% to 65% (Todd et al., 2015; Harris et al., 2017; Waldrop et al., 2019; Babic-Bozovic et al., 2021). The comparatively high diagnostic rate of 65.8% achieved in the present study can be attributed to several factors. Patients referred to the two tertiary care centers were a highly selected cohort with a strong clinical suspicion of underlying genetic etiology. Additionally, most patients had infantile-onset muscle weakness. The inclusion of the MLPA-negative DMD subgroup, projected to have a high diagnosis rate, significantly contributed to the overall performance. Moreover, unlike the practice of extensive prior gene panels or serial gene testing in many other countries, WES was used as an early diagnostic step. Consequently, WES revealed more positive findings than conventional approaches, increasing total diagnostic yield.

A drawback of early WES testing is its inability to identify the underlying genetic defects in two common adult-onset inherited myopathies: congenital myotonic dystrophy type 1 (CTD1) from *DMPK* repeat expansions and facioscapulohumeral muscular dystrophy (FSHD) due to contracted *D4Z4* repeats on chromosome 4q35. These conditions account for 10%–35% and 6%–10% of adult hereditary myopathies, ranking after DMD (Cotta et al., 2017; Pagola-Lorz et al., 2019). This study excluded FSHD suspects lacking molecular confirmation. Though none of the cohorts showed CTD1-specific symptoms, early-onset forms can resemble other myopathies. Omitting confirmed CTD1 and FSHD cases may artificially raise the apparent WES diagnostic yield.

Once a molecular diagnosis was established, muscle biopsies were avoided. Identifying DMD point mutations enabled steroid treatment initiation, while detecting a nonsense mutation qualified the patient for Ataluren therapy for premature stop codon readthrough (Richards et al., 2015). The COLQ congenital myasthenic syndrome patient may also receive targeted treatment based on the genetic analysis (Finsterer and Stöllberger, 2019). These examples demonstrate WES conclusion of the diagnostic process and allowing precision therapies.

Most patients in the MLPA-negative DMD group were successfully diagnosed. In some cases, WES revealed alternative inherited MD diagnoses, consistent with past research in DMD-resembling patients (Luce et al., 2018). Our negative MLPA DMD subgroup had no causal variants in other genes. Moving forward, targeted NGS may be considered for DMD-indicated patients with negative MLPA.

Accumulating evidence shows NGS technologies, chiefly targeted NGS and WES, confer high clinical value and shorten diagnostic times and expenses (Chae et al., 2015). Still, consensus pediatric neuromuscular genetic testing guidelines are lacking. We utilized first-line WES since most cases lacked clinical indications of specific disorders, excluding the MLPA-negative DMD group. Targeted NGS could be an alternative approach here. Limited gene panels risk omitting disorders beyond the initial differential. Further studies should evaluate early WES cost-effectiveness in this context.

While WES provides a comprehensive view of a patient’s coding genome, certain limitations exist. WES is unable to reliably detect copy number variations, triplet repeat expansions, or mutations in noncoding regions (Meienberg et al., 2016). Some technical limitations may be addressed as sequencing and bioinformatic techniques develop (Belkadi et al., 2015). Additionally, WES generates a large amount of data, requiring robust data storage capabilities and advanced bioinformatic knowledge for proper analysis and interpretation (Dewey et al., 2014). Ongoing challenges include incidental findings, variants of unknown significance, and the complexities of assigning pathogenicity (Amendola et al., 2016).

The high diagnostic yield of WES in our study (65.2%) may be influenced by selection bias, as our cohort consisted of patients with a strong clinical suspicion of an underlying genetic etiology and a high pre-test probability of a genetic diagnosis. This is in contrast to some previous studies that reported lower diagnostic yields for WES, which may have included more heterogeneous patient populations and employed WES as a second-tier test after extensive targeted genetic testing (Ankala et al., 2015; Ghaoui et al., 2015; Reddy et al., 2017). The inclusion of the MLPA-negative DMD subgroup, which had a high expected diagnosis rate, may have further contributed to the increased diagnostic efficacy observed in our study. It is important to consider these factors when interpreting and comparing diagnostic yields across different studies and patient populations. Future research should aim to investigate the efficacy of WES in more diverse and less selected patient cohorts and to compare the diagnostic performance of WES as a first-tier *versus* second-tier test (Tan et al., 2017; Howell et al., 2018). This will provide a more comprehensive understanding of the true diagnostic potential of WES in pediatric neuromuscular disorders and help guide its optimal implementation in clinical practice.

## Conclusion

In conclusion, our study demonstrates the effectiveness of WES in identifying disease-causing variants in Taiwanese pediatric patients with clinically diagnosed CM and MD. We found a high diagnostic yield of 65.2%, with pathogenic or likely pathogenic variants identified in 30 out of 46 patients. The most frequently implicated genes were *ZSWIM6, DMD, SYNE1, ATRX,* and *COL6A1*, which is consistent with previous studies on pediatric neuromuscular disorders (Dowling et al., 2018; Benarroch et al., 2020). Our results highlight the potential of WES to identify rare diseases that may present with overlapping phenotypes or atypical manifestations, and support the integration of WES into the diagnostic algorithm for pediatric neuromuscular disorders. The integration of WES into clinical practice has the potential to improve patient care and outcomes by enabling precise diagnoses, personalized management, and informed family planning.

## Data Availability

The datasets for this article are not publicly available due to concerns regarding participant/patient anonymity. Requests to access the datasets should be directed to the corresponding authors.

## References

[B1] Amendola L. M. Jarvik G. P. Leo M. C. McLaughlin H. M. Akkari Y. Amaral M. D. (2016). Performance of ACMG-AMP variant-interpretation guidelines among nine laboratories in the clinical sequencing exploratory research consortium. Am. J. Hum. Genet. 99, 247–276. 10.1016/j.ajhg.2016.06.001 27392081 PMC5005465

[B2] Ankala A. da Silva C. Gualandi F. Ferlini A. Bean L. J. H. Collins C. (2015). A comprehensive genomic approach for neuromuscular diseases gives a high diagnostic yield. Ann. Neurol. 77 (2), 206–214. 10.1002/ana.24303 25380242

[B3] Babic-Bozovic I. Dovc Drnovsek M. Cankar K. Meznaric M. Osredkar D. Peterlin B. (2021). Diagnostic yield of exome sequencing in myopathies: experience of a Slovenian tertiary centre. PLoS One 16, e0252953. 10.1371/journal.pone.0252953 34106991 PMC8189452

[B4] Bajaj K. Madhusudhan M. S. Adkar B. V. Chakrabarti P. Ramakrishnan C. Sali A. (2007). Stereochemical criteria for prediction of the effects of proline mutations on protein stability. PLoS Comput. Biol. 3 (12), e241. 10.1371/journal.pcbi.0030241 18069886 PMC2134964

[B5] Belkadi A. Bolze A. Itan Y. Cobat A. Vincent Q. B. Antipenko A. (2015). Whole-genome sequencing is more powerful than whole-exome sequencing for detecting exome variants. Proc. Natl. Acad. Sci. U. S. A. 112, 5473–5478. 10.1073/pnas.1418631112 25827230 PMC4418901

[B6] Benarroch L. Bonne G. Rivier F. Hamroun D. (2020). The 2021 version of the gene table of neuromuscular disorders (nuclear genome). Neuromuscul. Disord. 30, 1008–1048. 10.1016/j.nmd.2020.11.009 33257164

[B7] Birnkrant D. J. Bushby K. Bann C. M. Apkon S. D. Blackwell A. Brumbaugh D. (2018). Diagnosis and management of Duchenne muscular dystrophy, part 1: diagnosis, and neuromuscular, rehabilitation, endocrine, and gastrointestinal and nutritional management. Lancet Neurol. 17 (3), 251–267. 10.1016/S1474-4422(18)30024-3 29395989 PMC5869704

[B8] Böhm J. Vasli N. Malfatti E. Le Gras S. Feger C. Jost B. (2013). An integrated diagnosis strategy for congenital myopathies. PLoS One 8, e67527. 10.1371/journal.pone.0067527 23826317 PMC3691193

[B9] Butterfield R. J. Foley A. R. Dastgir J. Asman S. Dunn D. M. Zou Y. (2013). Position of glycine substitutions in the triple helix of COL6A1, COL6A2, and COL6A3 is correlated with severity and mode of inheritance in collagen VI myopathies. Hum. Mutat. 34 (11), 1558–1567. 10.1002/humu.22429 24038877 PMC4520221

[B10] Chae J. H. Kwak Y. Park H. C. Lim B. C. Zhang Q. Eun S. H. (2015). Utility of next generation sequencing in genetic diagnosis of early onset neuromuscular disorders. J. Med. Genet. 52, 208–216. 10.1136/jmedgenet-2014-102819 25635128

[B11] Cotta A. Paim J. F. Carvalho E. da-Cunha-Júnior A. L. F. Navarro M. M. Valicek J. (2017). The relative frequency of common neuromuscular diagnoses in a reference center. Arq. Neuropsiquiatr. 75 (11), 789–795. 10.1590/0004-282X20170151 29236822

[B12] Dewey F. E. Grove M. E. Pan C. Goldstein B. A. Bernstein J. A. Chaib H. (2014). Clinical interpretation and implications of whole-genome sequencing. JAMA 311, 1035–1045. 10.1001/jama.2014.1717 24618965 PMC4119063

[B13] Doniger S. W. Kim H. S. Swain D. Corcuera D. Williams M. Yang S. P. (2008). A catalog of neutral and deleterious polymorphism in yeast. PLoS Genet. 4 (8), e1000183. Published 2008 Aug 29. 10.1371/journal.pgen.1000183 18769710 PMC2515631

[B14] Dowling J. J. D Gonorazky H. Cohn R. D. Campbell C. (2018). Treating pediatric neuromuscular disorders: the future is now. Am. J. Med. Genet. A 176 (4), 804–841. 10.1002/ajmg.a.38418 28889642 PMC5900978

[B15] Ferrer-Costa C. Orozco M. de la Cruz X. (2002). Characterization of disease-associated single amino acid polymorphisms in terms of sequence and structure properties. J. Mol. Biol. 315 (4), 771–786. 10.1006/jmbi.2001.5255 11812146

[B16] Finsterer J. Stöllberger C. (2019). Congenital myasthenic syndromes. Orphanet J. Rare Dis. 14, 57. 10.1186/s13023-019-1025-5 30808424 PMC6390566

[B17] Flanigan K. M. Dunn D. M. von Niederhausern A. Soltanzadeh P. Gappmaier E. Howard M. T. (2009). Mutational spectrum of DMD mutations in dystrophinopathy patients: application of modern diagnostic techniques to a large cohort. Hum. Mutat. 30 (12), 1657–1666. 10.1002/humu.21114 19937601 PMC3404892

[B18] Gao L. Uzun Y. Gao P. He B. Ma X. Wang J. (2018). Identifying noncoding risk variants using disease-relevant gene regulatory networks. Nat. Commun. 9 (1), 702. Published 2018 Feb 16. 10.1038/s41467-018-03133-y 29453388 PMC5816022

[B19] Ghaoui R. Cooper S. T. Lek M. Jones K. Corbett A. Reddel S. W. (2015). Use of whole-exome sequencing for diagnosis of limb-girdle muscular dystrophy: outcomes and lessons learned. JAMA Neurol. 72 (12), 1424–1432. 10.1001/jamaneurol.2015.2274 26436962

[B20] Harris E. Topf A. Barresi R. Hudson J. Powell H. Tellez J. (2017). Exome sequences versus sequential gene testing in the UK highly specialised Service for Limb Girdle Muscular Dystrophy. Orphanet J. Rare Dis. 12, 151. 10.1186/s13023-017-0699-9 28877744 PMC5588739

[B21] Howell K. B. Eggers S. Dalziel K. Riseley J. Mandelstam S. Myers C. T. (2018). A population-based cost-effectiveness study of early genetic testing in severe epilepsies of infancy. Epilepsia 59 (6), 1177–1187. 10.1111/epi.14087 29750358 PMC5990455

[B22] Kang P. B. Morrison L. Iannaccone S. T. Graham R. J. Bönnemann C. G. Rutkowski A. (2015). Evidence-based guideline summary: evaluation, diagnosis, and management of congenital muscular dystrophy: report of the guideline development subcommittee of the American academy of neurology and the practice issues review panel of the American association of neuromuscular & electrodiagnostic medicine. Neurology 84 (13), 1369–1378. 10.1212/WNL.0000000000001416 25825463 PMC4388744

[B23] Lampe A. K. Bushby K. M. (2005). Collagen VI related muscle disorders. J. Med. Genet. 42 (9), 673–685. 10.1136/jmg.2002.002311 16141002 PMC1736127

[B24] Luce L. N. Villaverde C. Jacob G. Ferrer M. Szijan I. Giliberto F. (2018). Small mutation screening in the DMD gene by whole exome sequencing of an argentine Duchenne/Becker muscular dystrophies cohort. Neuromuscul. Disord. 28, 986–995. 10.1016/j.nmd.2018.08.012 30342905

[B25] Meienberg J. Bruggmann R. Oexle K. Matyas G. (2016). Clinical sequencing: is WGS the better WES? Hum. Genet. 135, 359–362. 10.1007/s00439-015-1631-9 26742503 PMC4757617

[B26] Mercuri E. Bönnemann C. G. Muntoni F. (2019). Muscular dystrophies. Lancet 394, 2025–2038. 10.1016/S0140-6736(19)32910-1 31789220

[B27] Miller M. P. Kumar S. (2001). Understanding human disease mutations through the use of interspecific genetic variation. Hum. Mol. Genet. 10 (21), 2319–2328. 10.1093/hmg/10.21.2319 11689479

[B28] Naghipoor K. Khosravi T. Oladnabi M. (2023). Whole exome sequencing identifies a novel variant in the COL12A1 gene in a family with Ullrich congenital muscular dystrophy 2. Mol. Biol. Rep. 50 (9), 7427–7435. 10.1007/s11033-023-08644-6 37458870

[B29] Natsuga K. Nishie W. Shinkuma S. Arita K. Nakamura H. Ohyama M. (2010). Plectin deficiency leads to both muscular dystrophy and pyloric atresia in epidermolysis bullosa simplex. Hum. Mutat. 31 (10), E1687–E1698. 10.1002/humu.21330 20665883 PMC3023027

[B30] Ng P. C. Henikoff S. (2003). SIFT: predicting amino acid changes that affect protein function. Nucleic Acids Res. 31 (13), 3812–3814. 10.1093/nar/gkg509 12824425 PMC168916

[B31] North K. N. Wang C. H. Clarke N. Jungbluth H. Vainzof M. Dowling J. J. (2014). Approach to the diagnosis of congenital myopathies. Neuromuscul. Disord. 24, 97–116. 10.1016/j.nmd.2013.11.003 24456932 PMC5257342

[B32] Okubo M. Minami N. Goto K. Goto Y. Noguchi S. Nishino I. (2016). Genetic diagnosis of Duchenne/Becker muscular dystrophy using next-generation sequencing: validation analysis of DMD mutations. J. Hum. Genet. 61 (6), 483–489. 10.1038/jhg.2016.7 26911353 PMC4931045

[B33] Pagola-Lorz I. Villar-Quiles R. N. Soriano-González J. Torné L. Elizalde-Beiras I. Garcia-Solaesa V. (2019). Epidemiological study and genetic characterization of inherited muscle diseases in a northern Spanish region. Orphanet J. Rare Dis. 14, 276. 10.1186/s13023-019-1227-x 31791368 PMC6889463

[B34] Palmer E. E. Kumar R. Gordon C. T. Shaw M. Hubert L. Carroll R. (2017). A recurrent *de novo* nonsense variant in ZSWIM6 results in severe intellectual disability without frontonasal or limb malformations. Am. J. Hum. Genet. 101 (6), 995–1005. 10.1016/j.ajhg.2017.10.009 29198722 PMC5812890

[B35] Reddy H. M. Cho K. A. Lek M. Estrella E. Valkanas E. Jones M. D. (2017). The sensitivity of exome sequencing in identifying pathogenic mutations for LGMD in the United States. J. Hum. Genet. 62 (2), 243–252. 10.1038/jhg.2016.116 27708273 PMC5266644

[B36] Richards S. Aziz N. Bale S. Bick D. Das S. Gastier-Foster J. (2015). Standards and guidelines for the interpretation of sequence variants: a joint consensus recommendation of the American College of medical genetics and genomics and the association for molecular pathology. Genet. Med. 17, 405–424. 10.1038/gim.2015.30 25741868 PMC4544753

[B37] Sewry C. A. Laitila J. M. Wallgren-Pettersson C. (2019). Nemaline myopathies: a current view. J. Muscle Res. Cell Motil. 40 (2), 111–126. 10.1007/s10974-019-09519-9 31228046 PMC6726674

[B38] Straub V. Murphy A. Udd B. LGMD workshop study group (2018). 229th ENMC international workshop: limb girdle muscular dystrophies - nomenclature and reformed classification Naarden, The Netherlands, 17-19 March 2017. Neuromuscul. Disord. 28 (8), 702–710. 10.1016/j.nmd.2018.05.007 30055862

[B39] Tan T. Y. Dillon O. J. Stark Z. Schofield D. Alam K. Shrestha R. (2017). Diagnostic impact and cost-effectiveness of whole-exome sequencing for ambulant children with suspected monogenic conditions. JAMA Pediatr. 171 (9), 855–862. 10.1001/jamapediatrics.2017.1755 28759686 PMC5710405

[B40] Tavtigian S. V. Greenblatt M. S. Harrison S. M. Nussbaum R. L. Prabhu S. A. Boucher K. M. (2018). Modeling the ACMG/AMP variant classification guidelines as a Bayesian classification framework. Genet. Med. 20 (9), 1054–1060. 10.1038/gim.2017.210 29300386 PMC6336098

[B41] Tawil R. Kissel J. T. Heatwole C. Pandya S. Gronseth G. Benatar M. (2015). Evidence-based guideline summary: evaluation, diagnosis, and management of facioscapulohumeral muscular dystrophy: report of the guideline development, dissemination, and implementation subcommittee of the American academy of neurology and the practice issues review panel of the American association of neuromuscular & electrodiagnostic medicine. Neurology 85 (4), 357–364. 10.1212/WNL.0000000000001783 26215877 PMC4520817

[B42] Todd E. J. Yau K. S. Ong R. Slee J. McGillivray G. Barnett C. P. (2015). Next generation sequencing in a large cohort of patients presenting with neuromuscular disease before or at birth. Orphanet J. Rare Dis. 10, 148. 10.1186/s13023-015-0364-0 26578207 PMC4650299

[B43] Tuffery-Giraud S. Béroud C. Leturcq F. Yaou R. B. Hamroun D. Michel-Calemard L. (2009). Genotype-phenotype analysis in 2,405 patients with a dystrophinopathy using the UMD-DMD database: a model of nationwide knowledgebase. Hum. Mutat. 30 (6), 934–945. 10.1002/humu.20976 19367636

[B44] Twigg S. R. Ousager L. B. Miller K. A. Zhou Y. Elalaoui S. C. Sefiani A. (2016). Acromelic frontonasal dysostosis and ZSWIM6 mutation: phenotypic spectrum and mosaicism. Clin. Genet. 90 (3), 270–275. 10.1111/cge.12721 26706854 PMC5025718

[B45] Valencia C. A. Ankala A. Rhodenizer D. Bhide S. Littlejohn M. R. Keong L. M. (2013). Comprehensive mutation analysis for congenital muscular dystrophy: a clinical PCR-based enrichment and next-generation sequencing panel. PLoS One 8, e53083. 10.1371/journal.pone.0053083 23326386 PMC3543442

[B46] Waldrop M. A. Sulek M. Semeraro M. L. Sites E. Bartholomew D. Tsao C. Y. (2019). Diagnostic utility of whole exome sequencing in the neuromuscular clinic. Neuropediatrics 50, 96–102. 10.1055/s-0039-1677734 30665247

[B47] Winter L. Wiche G. (2013). The many faces of plectin and plectinopathies: pathology and mechanisms. Acta Neuropathol. 125 (1), 77–93. 10.1007/s00401-012-1026-0 22864774

[B48] Witting N. Werlauff U. Duno M. Vissing J. (2017). Phenotypes, genotypes, and prevalence of congenital myopathies older than 5 years in Denmark. Neurol. Genet. 3 (2), e140. Published 2017 Mar 21. 10.1212/NXG.0000000000000140 28357410 PMC5362145

