# Supplementary material for: Application of whole exome sequencing in the diagnosis of muscular disorders: a study of Taiwanese pediatric patients
Source: Front Genet. 2024 May 15;15:1365729. doi: 10.3389/fgene.2024.1365729 (PMC11137626; doi:10.3389/fgene.2024.1365729)
Supplement: Supplementary file 1 [file DataSheet1.pdf]

Supplementary Table S1. Conservation analysis of identified variants in the Congenital Myopathy (CM) group. N/A: not available

Gene	Nucleotide change	Amino acid change	Conservation status	Methods
ZSWIM6	c.532_533insT	p.Ala178Valfs*76	Highly conserved	UCSC Genome Browser (100 vertebrates basewise conservation by PhyloP)
OCRL	c.1174+1G>T	Splice site	Highly conserved	UCSC Genome Browser (100 vertebrates basewise conservation by PhyloP)
ATRX	c.736C>T	p.Arg246Cys	Highly conserved	UCSC Genome Browser (100 vertebrates basewise conservation by PhyloP), CDD (conserved domain)
COL12A1	c.5894G>A	p.Gly1965Glu	Moderately conserved	UCSC Genome Browser (100 vertebrates basewise

Gene	Nucleotide change	Amino acid change	Conservation status	Methods
				conservation by PhyloP)
SYNE1	c.5627G>A	p.Ser1876Asn	Poorly conserved	UCSC Genome Browser (100 vertebrates basewise conservation by PhyloP)
NIPBL	c.53G>C	p.Ser18Thr	Highly conserved	UCSC Genome Browser (100 vertebrates basewise conservation by PhyloP), CDD (conserved domain)
VARS	c.3596G>A	p.Arg1199Gin	Highly conserved	UCSC Genome Browser (100 vertebrates basewise conservation by PhyloP), CDD (conserved domain)
CNTNAP1	c.3361C>T	p.Arg1121*	Highly conserved	UCSC Genome Browser (100

Gene	Nucleotide change	Amino acid change	Conservation status	Methods
				vertebrates basewise conservation by PhyloP)
TUBB4A	c.1172G>A	p.Arg391His	Highly conserved	UCSC Genome Browser (100 vertebrates basewise conservation by PhyloP), CDD (conserved domain)
OFD1	c.2del	p.Met1	N/A	N/A
COL6A1	c.850G>A	p.Gly284Arg	Highly conserved	UCSC Genome Browser (100 vertebrates basewise conservation by PhyloP), CDD (conserved domain)
ATN1	c.1449C>A	p.His483Gln	Moderately conserved	UCSC Genome Browser (100 vertebrates basewise conservation by PhyloP)

Gene	Nucleotide change	Amino acid change	Conservation status	Methods
NEB	c.11606T>C, c.18800T>C	p.Tyr3869Tyr p.Ile6267Thr	Moderately conserved	UCSC Genome Browser (100 vertebrates basewise conservation by PhyloP)
AK9	c.3614G>T c.529G>T	p.Arg1205Ile p.Asp177Tyr	Poorly conserved	UCSC Genome Browser (100 vertebrates basewise conservation by PhyloP)
CHRNA1	c.257G>A	p.Arg86His	Highly conserved	UCSC Genome Browser (100 vertebrates basewise conservation by PhyloP), CDD (conserved domain)

Supplementary Table S2. Conservation analysis of identified variants in the Muscular Dystrophy (MD) group

Gene	Nucleotide change	Amino acid change	Conservation status	Methods
ZSWIM6	c.481delC	p.Ala161fs	Highly conserved	UCSC Genome Browser (100 vertebrates basewise conservation by PhyloP)
SLC37A4	c.1042_1043delCT<br>c.898C>T	p.Leu348fs*53<br>p.Arg300Cys	Moderately conserved	UCSC Genome Browser (100 vertebrates basewise conservation by PhyloP)
PLEC	c.9343C>T<br>c.13192G>A	p.Arg3115Cys<br>p.Ala4398Thr	Moderately conserved	UCSC Genome Browser (100 vertebrates basewise conservation by PhyloP)
GJB2	c.109G>A	p.Val37Ile	Poorly conserved	UCSC Genome Browser (100 vertebrates basewise conservation by PhyloP)
SH3TC2	c.1817_1818del	p.Glu606ValfsTer2	Moderately conserved	UCSC Genome Browser (100 vertebrates basewise conservation by PhyloP)
ARIDG	c.1717dup	p.Trp573LeufsTer45	Highly conserved	UCSC Genome Browser (100 vertebrates basewise

Gene	Nucleotide change	Amino acid change	Conservation status	Methods
				conservation by PhyloP), CDD (conserved domain)
ACSL4	c.1126-4T>C	Splice site	Highly conserved	UCSC Genome Browser (100 vertebrates basewise conservation by PhyloP)
MCM3AP	c.5383G>A<br>c.998G>T	p.Glu1795Lys<br>p.Cys333Phe	Moderately conserved	UCSC Genome Browser (100 vertebrates basewise conservation by PhyloP)
FUK	c.428C>T<br>c.1341+1G>T	p.Pro143Leu<br>Splice site	Highly conserved	UCSC Genome Browser (100 vertebrates basewise conservation by PhyloP), CDD (conserved domain)
STXBP1	c.1706C>G	p.Ser569Cys	Highly conserved	UCSC Genome Browser (100 vertebrates basewise conservation by PhyloP), CDD (conserved domain)
BPTF	c.205G>C	p.Gly69Arg	Highly conserved	UCSC Genome Browser (100 vertebrates basewise

Gene	Nucleotide change	Amino acid change	Conservation status	Methods
				conservation by PhyloP), CDD (conserved domain)
PRS6KA3	c.2182C>T	p.Gln728*	Highly conserved	UCSC Genome Browser (100 vertebrates basewise conservation by PhyloP)

Supplementary Table S3. Conservation analysis of identified variants in the MLPA Negative DMD group

Gene	Nucleotide change	Amino acid change	Conservation status	Methods
DMD	c.7354G>T	p.Glu2452*(E2452*)	Highly conserved	UCSC Genome Browser (100 vertebrates basewise conservation by PhyloP)
DMD	c.7993A>G	p.Asn2665Asp	Highly conserved	UCSC Genome Browser (100 vertebrates basewise conservation by PhyloP), CDD (conserved domain)

Gene	Nucleotide change	Amino acid change	Conservation status	Methods
DMD	c.5190G>C	p.Lys1730Asn	Highly conserved	UCSC Genome Browser (100 vertebrates basewise conservation by PhyloP), CDD (conserved domain)

Supplementary Table S4. Previously reported mutations in genes associated with muscular disorders identified in this study

Gene	Nucleotide change	Amino acid change	Associated phenotype	References
DMD	c.7354G>T	p.Glu2452*(E2452*)	Duchenne muscular dystrophy	13
DMD	c.7993A>G	p.Asn2665Asp	Duchenne muscular dystrophy	14
DMD	c.5190G>C	p.Lys1730Asn	Duchenne muscular dystrophy	15
PLEC	c.9343C>T	p.Arg3115Cys	Epidermolysis bullosa simplex with muscular dystrophy	16

Gene	Nucleotide change	Amino acid change	Associated phenotype	References
PLEC	c.13192G>A	p.Ala4398Thr	Epidermolysis bullosa simplex with muscular dystrophy	17
COL12A1	c.5894G>A	p.Gly1965Glu	Bethlem myopathy 2/ Ullrich congenital muscular dystrophy	18
COL6A1	c.850G>A	p.Gly284Arg	Bethlem myopathy 1 / Ullrich congenital muscular dystrophy	19,20
